# Neighborhood Effects on Heat Deaths: Social and Environmental Predictors of Vulnerability in Maricopa County, Arizona

**DOI:** 10.1289/ehp.1104625

**Published:** 2012-11-16

**Authors:** Sharon L. Harlan, Juan H. Declet-Barreto, William L. Stefanov, Diana B. Petitti

**Affiliations:** 1 School of Human Evolution and Social Change, Arizona State University, Tempe, Arizona, USA; 2 Science Applications Research and Development, Jacobs/Engineering Science and Contract Group, NASA Johnson Space Center, Houston, Texas, USA; 3 Department of Biomedical Informatics, Arizona State University, Scottsdale, Arizona, USA

**Keywords:** climate, GIS, heat mortality, neighborhoods, remote sensing, vulnerability

## Abstract

Background: Most heat-related deaths occur in cities, and future trends in global climate change and urbanization may amplify this trend. Understanding how neighborhoods affect heat mortality fills an important gap between studies of individual susceptibility to heat and broadly comparative studies of temperature–mortality relationships in cities.

Objectives: We estimated neighborhood effects of population characteristics and built and natural environments on deaths due to heat exposure in Maricopa County, Arizona (2000–2008).

Methods: We used 2000 U.S. Census data and remotely sensed vegetation and land surface temperature to construct indicators of neighborhood vulnerability and a geographic information system to map vulnerability and residential addresses of persons who died from heat exposure in 2,081 census block groups. Binary logistic regression and spatial analysis were used to associate deaths with neighborhoods.

Results: Neighborhood scores on three factors—socioeconomic vulnerability, elderly/isolation, and unvegetated area—varied widely throughout the study area. The preferred model (based on fit and parsimony) for predicting the odds of one or more deaths from heat exposure within a census block group included the first two factors and surface temperature in residential neighborhoods, holding population size constant. Spatial analysis identified clusters of neighborhoods with the highest heat vulnerability scores. A large proportion of deaths occurred among people, including homeless persons, who lived in the inner cores of the largest cities and along an industrial corridor.

Conclusions: Place-based indicators of vulnerability complement analyses of person-level heat risk factors. Surface temperature might be used in Maricopa County to identify the most heat-vulnerable neighborhoods, but more attention to the socioecological complexities of climate adaptation is needed.

Deaths caused by extremely hot weather are a major public health concern and temperature–mortality relationships in many cities have been intensively studied over the past decade (McMichael et al. 2008). Global climate models predict higher temperatures and more frequent, longer, and more intense extreme heat events over most of the world, and this has greatly increased estimates of future deaths due to heat (Intergovernmental Panel on Climate Change 2007). Most heat-related deaths occur in cities (Kovats and Koppe 2005), and this trend is likely to continue for several socio-environmental reasons: *a*) Cities in many types of climate regimes experience heat waves (Anderson and Bell 2011); *b*) cities, especially in developing nations of Asia and Africa, are growing rapidly (United Nations Population Fund 2007); *c*) large numbers of vulnerable populations, such as the poor, homeless, and elderly, reside in cities; and *d*) cities are warmer than surrounding rural areas because of the urban heat island effect (Heisler and Brazel 2010).

Many studies on urban heat-related mortality examine individual-specific risk factors. Commonly identified physiological risks, summarized by Balbus and Malina (2009), include advanced or young age, underlying disease, disability, and pregnancy. Cardiovascular disease and several other illnesses are risk factors for heat-related death (McMichael et al. 2006). Deaths from heat exposure also occur among people who lack access to cool environments or are physically active in hot weather (Kilbourne et al. 1982). Living in poverty is a key individual risk factor for death related to heat because it decreases the odds of access to medical care and protective resources (Balbus and Malina 2009).

Neighborhoods are “ecological units nested within successively larger communities” [Sampson et al. 2002 (p. 445)] and neighborhood effects on human development and life cycle events, individual behavior, social outcomes, and health risks have long been studied in the social and health sciences (Brooks-Gunn et al. 1993; Pickett and Pearl 2001; Sampson et al. 2002). Many indicators of poor health, such as low birth weight (Debbink and Bader 2011), obesity (Mujahid et al. 2008), and coronary heart disease (Diez-Roux 2001) are spatially clustered within neighborhoods. Socioeconomic context is important because many poor neighborhoods lack institutional capacities for education, health care, and employment (Wilson 1987) and have poor quality housing. Many minorities live in low-income areas with high levels of social isolation and concentrated disadvantage (Sampson 2009). Lochner et al. (2002) found that economic deprivation and low levels of social capital—defined as trust, social ties, and reciprocity—were associated with higher all-cause mortality in Chicago, Illinois, neighborhoods.

There are numerous suspected pathways through which living in a poor neighborhood could lead to higher risks of heat-related deaths. An emergent literature on community and environmental heat risk factors has examined differences in socioeconomic and social network variables (Klinenberg 2002), the built environment (Kovats and Koppe 2005; O’Neill et al. 2005), and the biophysical environment (Harlan et al. 2006; Ruddell et al. 2010). Studies in St. Louis, Missouri (Smoyer 1998), Chicago, Illinois (Johnson et al. 2012; Klinenberg 2002), and Philadelphia, Pennsylvania (Johnson et al. 2009) have investigated differences in deaths by neighborhood during heat waves. Although such studies of neighborhood-specific deaths are rare, investigations of fine-scale variation in social and environmental neighborhood contexts within cities may fill an important gap between studies of individual susceptibility to heat and broadly comparative studies of temperature–mortality relationships in cities across different climate regimes and geographic locations, such as McMichael et al. (2008).

In the present study, we investigated neighborhood effects on heat exposure deaths in Maricopa County, Arizona, over a 9-year period (2000–2008). Extremely high temperatures occur almost daily for 6 months a year in this desert climate. The decedents were identified by the county health department using a surveillance system designed specifically to detect deaths caused by or related to environmental (weather-related) heat. Our question was “What characteristics of urban neighborhoods affect the risk of residents dying from extreme heat?”

We traced neighborhood effects through two pathways that are connected to individual health outcomes. Neighborhood compositional effects are aggregated population characteristics, especially those related to socioeconomic status and age, which increase the risks of heat mortality. Neighborhood contextual effects are characteristics of the built and natural environments in which people perform their daily activities. Contextual influences on urban heat mortality, such as land cover and microclimates, have been studied much less than population characteristics, but they may be important markers for places that are vulnerable to heat.

Found in metropolitan regions, the strongly increasing temperature gradient from rural areas to central cities is known as the urban heat island effect (Heisler and Brazel 2010). Diurnal temperature cycles vary substantially between neighborhoods, and differences in neighborhood land cover characteristics—vegetation, impervious surface, bare soil, and water—and building structures are drivers of local temperatures (Buyantuyev and Wu 2010). More vegetation lowers local temperature because green areas—trees, other plants, and lawns in yards and parks—cool the air and ground surfaces through shading and evapotranspiration. Studies have documented neighborhood variations in land cover and/or temperature within Phoenix (Buyantuyev and Wu 2010; Jenerette et al. 2011), Detroit, Michigan (Zhang et al. 2011), Philadelphia, Pennsylvania (Johnson et al. 2009), and Baltimore, Maryland (Huang et al. 2011).

The amount of vegetation cover and land surface temperature (e.g., the temperature one would sense by touching the land surface) are biophysical properties of the outdoor neighborhood environment that are associated with human heat stress (Harlan et al. 2006) and heat-related deaths (Johnson and Wilson 2009; Johnson et al. 2012) in specific geographic contexts. Remotely sensed data from satellites provides synoptic spatial coverage of vegetation and land surface temperature for entire neighborhoods, but the temporal coverage of a given scene is limited to a single point in time, much like a snapshot from a hand-held camera. On the other hand, air temperature—measured at approximately 2 m above the land surface—is monitored continuously at weather stations, but their proximity to residential neighborhoods is highly variable. Heat mortality studies that use air temperature measurements from a single station or an average temperature of several stations ignore spatial variability.

Our study used two approaches to identify heat-vulnerable neighborhoods. First, we quantified neighborhood thermal properties with spatially continuous remotely sensed metrics to provide measures of difference in neighborhood environmental contexts that cannot be obtained from spatially discontinuous air temperature measurements. Second, we used the cumulative heat vulnerability index (HVI) that Reid et al. (2009) developed for census tracts in the United States and subsequently applied to predicting morbidity and mortality in some ZIP codes in five states (Reid et al. 2012). We recalculated and mapped Reid’s index at the finer spatial resolution of census block groups in the urbanized area of Maricopa County. HVI is an index of vulnerability that sums aggregated neighborhood population characteristics, prevalence of air conditioning (AC), and amount of vegetation cover in order to cumulate the known risk factors for heat mortality.

The rationale for mapping vulnerability is to locate spatially the distributions of social disadvantages and environmental hazards so that effective adaptive strategies can be developed for populations that are most likely to be exposed to them (Cutter and Finch 2008). Mapping may help policy makers identify areas of cities that are at high risk for heat-related mortality, are in need of preventive actions during hot weather, and are high priorities for environmental modifications.

One of the present study’s original contributions was to perform analyses that evaluate the relationship between the HVI and heat-associated mortality in Maricopa County residential neighborhoods. Another contribution was to assess differences among models that use neighborhood HVI or land surface temperature (hereafter referred to as surface temperature) to predict the odds of heat exposure deaths. The sparse literature leads us to expect that neighborhood effects of social variables, green spaces, and temperature are all positively related to heat mortality.

## Methods

*Study area.* The population center of Maricopa County is the Phoenix metropolitan area, which comprises > 20 contiguous municipalities and three tribal communities. Phoenix is the largest city, but several other medium and large cities are also located within our study area. The area’s population numbered 3.8 million people in 2010.

Chronically hot weather causes many heat fatalities in the desert southwest (mean daily high temperature in Phoenix = 39.4°C during May–September 2000–2008). Arizona has led the nation in deaths from heat exposure (Centers for Disease Control and Prevention 2006), and there is a strong positive temperature–mortality relationship in Maricopa County (Yip et al. 2008). In some Phoenix neighborhoods, the heat stress index was above the National Oceanic and Atmospheric Administration danger threshold for 18% of all hours during summer 2003 (Harlan et al. 2006).

There is no single agreed-upon definition of neighborhood, despite widespread interest in neighborhood effects (Sampson et al. 2002). Reid et al. (2009), Smoyer (1998), and Johnson et al. (2009) used census tract boundaries in their studies of intraurban heat vulnerability. We used block group boundaries from the 2000 U.S. Census (2002) to define neighborhoods for our study because they are subdivisions of larger census tracts and therefore more socially and ecologically homogeneous than tracts (see also Johnson et al. 2012). We included 2,081 block groups in our analysis after eliminating 19 with < 10 residents, 11 with missing data on any variable in the analysis, and 2 containing only institutionalized populations in a state hospital or juvenile corrections facility.

*Neighborhood composition.* We considered variables from the 2000 U.S. Census used by Reid et al. (2009) to derive a national literature-based HVI. Selected variables showed strong relationships with individual heat mortality, specifically, percentages of populations that were ethnic minorities, were living below the poverty line, had < high school education, were elderly (≥ 65 years of age), were living alone, and were both elderly and living alone. In addition, our analysis included percentages of Latino immigrants, defined as foreign-born Spanish-only speakers, because they made up 10.4% of Maricopa County’s population in 2000 and may be vulnerable to environmental health problems in part due to conditions in their neighborhoods (Grineski et al. 2007). We did not use diabetes prevalence when constructing our block-group-level HVI because diabetes data were only available at the state level.

*Neighborhood context.* We defined neighborhood contextual effects related to heat vulnerability as the cooling capacities of indoor (i.e., the prevalence of AC) and outdoor (i.e., vegetation and surface temperature) environments. These measures represent average differences in neighborhood environments to which people are exposed that may influence exposure to heat but do not provide information on individual-level exposure to heat.

The Maricopa County Tax Assessor records whether residential parcels have central AC or an evaporative cooler, an apparatus that cools home interiors by evaporating water. Evaporative coolers are used in some older and low-income homes in Arizona instead of AC, but they are not effective when the drier air of June and July transitions to the higher humidity of the August monsoon in central Arizona (Kalkstein and Kalkstein 2004). We spatially joined the Tax Assessor’s 2010 parcel registry using a geographic information system (GIS) to calculate the percentage of single-family houses in each census block group that did not have central AC or an evaporative cooler (these are not reported separately in the data source). The available data do not account for houses with window units only or for AC/cooler availability in apartment complexes.

We calculated surface temperature and the normalized difference vegetation index (NDVI; Tucker 1979)—a numerical measure of green vegetation abundance at the pixel scale based on the light reflectance properties of vegetation—for each pixel in the study area from a Landsat scene (30 m resolution) acquired on 24 July 2000 at 1055 hours local time. NDVI and surface temperature pixel scores were aggregated to block group boundaries to measure mean outdoor thermal properties of neighborhoods. We also used NDVI and surface temperature SDs because the spatial variability of vegetation and temperatures within neighborhoods are important properties of neighborhoods’ thermal profiles (Jenerette et al. 2007).

We chose July measures because half the heat exposure deaths occurred during this month in each year of the study. In the Phoenix area, air and surface temperatures differ widely and consistently across neighborhoods, and differences are greater during the summer and even greater during heat waves (Harlan et al. 2006; Jenerette et al. 2011; Ruddell et al. 2010).

*Heat deaths.* The Maricopa County Department of Public Health (MCDPH) has a surveillance system specifically designed to identify heat-caused and heat-related deaths associated with weather (MCDPH 2011). Their system collects information from several sources: the Office of Medical Examiner case list, which identifies suspected heat-associated deaths referred to the Medical Examiner; the Arizona Department of Health Services, which identifies deaths with environmental heat mentioned on death certificates; local hospitals, which identify suspected heat-associated morbidity/mortality cases; and media reports of heat-related deaths. A daily search of the electronic death certificate database for multiple causes of death identifies conditions associated with environmental heat: *International Classification of Disease*, *Tenth Revision* codes (World Health Organization 2007) (i.e., X30, exposure to excessive natural heat; T67.X, effects of heat and light; P81.0, environmental hyperthermia of newborn) and key phrases in the text fields for causes and underlying causes of death (i.e., heat exposure, environ, exhaustion, sun, heat stress, heat stroke, hyperthermia). The surveillance system designated 455 deaths caused by or related to environmental heat exposure that occurred between 2000 and 2008 (annual mean = 50.6; SD = 11.6; range = 25–85). The age distribution of the decedents was as follows: 0–19 years (5.1%); 20–39 years (13.0%); 40–64 years (45.4%); 65–74 years (14.3%); ≥ 75 years (22.1%).

We geocoded 278 residential street addresses of these decedents and calculated the incidence of heat deaths for each block group. Other residential addresses were either outside Maricopa County or unknown. The MCDPH considers unknown street addresses in the county to be a marker of homelessness. We were able to geocode a place of injury address from the death certificate for 50 heat decedents with unknown residential street addresses in Maricopa County (presumed homeless). These deaths were analyzed separately.

*Analysis*. Means, SDs, and Pearson correlations for variables related to heat vulnerability are reported in Table 1. Variables were coded so that higher scores denote higher vulnerability. We performed a principal components analysis on the correlation matrix for the HVI variables using varimax rotation and standard statistical criteria, which yielded three principal factors (eigenvalues > 1.5; total explained common variance = 79.8%). Standardized scores for each factor (mean = 0; SD = 1) were assigned to block groups. Following Reid et al. (2009), each factor score was also divided into six equal increments of ± 1.0 SD and assigned integer values 1 (≥ 2 SD below mean) to 6 (≥ 2 SD above mean). HVI for block groups were calculated by summing the integer scores for all three factors [possible total scores of 3–18, actual scores ranged from 5–16 (median = 10; mean = 10.6; SD = 1.6)]. We mapped the index onto the study area and then overlaid point estimates of the residential addresses of decedents at the centroid of block groups.

**Table 1:** Means, SDs, and Pearson’s correlations for variables in the 2000 U.S. Census Maricopa County block groups (*n* = 2,081).

Variable	Ethnic minority	Latino immigrant	< Poverty line	No HS diploma	Age ≥ 65 years	Age ≥ 65 × living alone	Living alone	No AC/ cooler	Unvegetated area		Land surface temp	
									Mean	SD	Mean	SD
Mean	0.34	0.11	0.12	0.20	0.13	0.08	0.24	0.13	–0.21	–0.11	54.28	2.14
SD	0.26	0.12	0.13	0.19	0.17	0.09	0.14	0.22	0.07	0.06	1.94	1.19
Ethnic minority	1.00
Latino immigrant	0.81**	1.00
< Poverty line	0.74**	0.71**	1.00
No HS diploma	0.84**	0.80**	0.73**	1.00
≥ 65 years of age	–0.40**	–0.25**	–0.18**	–0.14**	1.00
≥ 65 years of age × living alone	–0.26**	–0.14**	–0.03	–0.02	0.88**	1.00
Living alone	–0.16**	–0.09**	0.10**	–0.60**	0.45**	0.63**	1.00
No AC/cooler	0.67*	0.64**	0.57**	0.68**	–0.16**	–0.04	–0.06	1.00
Unvegetated area, 24 July 2000 (mean)	0.16**	0.17**	0.22**	0.20**	0.00	0.02	0.04	0.15**	1.00
Unvegetated area, 24 July 2000 (SD)	0.10**	0.13**	0.11**	0.10**	–0.14**	–0.08**	–0.06**	0.16**	0.69**	1.00
Land surface temperature, 24 July 2000 (mean)	0.35**	0.32**	0.33**	0.37**	–0.11**	–0.06**	–0.08**	0.32**	0.78**	0.67**	1.00
Land surface temperature, 24 July 2000 (SD)	–0.08**	–0.13**	–0.07**	–0.06**	0.10**	0.04	0.00	–0.18*	–0.47**	–0.86**	–0.54**	1.00
HS, high school. *p ≤ 0.05. **p ≤ 0.01.																		

We estimated binary logistic regressions (SAS version 9.2; SAS Institute Inc., Cary, NC) to predict the odds that a block group was home to ≥ 1 decedents who died from heat exposure (1 = yes; 0 = no). There were no deaths in 88.6% (1,843) of the block groups (204 = 1 death; 31 = 2 deaths; 3 = 3 deaths). Because of the small numbers associated with a rare event (Cromley and McLafferty 2002), we did not attempt to model mortality rates. There were too few block groups with > 1 death to explain the variation in the number of deaths. Population size in 2000 (best available estimate for 2000–2008) was included in each model to control for the number of people at risk in each neighborhood.

To evaluate neighborhood effects on the odds of heat deaths, we tested eight models with different combinations of covariates: the HVI additive index score, each individual HVI factor score from the principal components analysis, all three HVI factor scores, percent of homes with no AC/coolers, land surface temperature, and a combination of all HVI factor scores and surface temperature. We used the Bayesian information criterion (BIC) to make pairwise comparisons between the binomial regression models. The BIC assesses model fit penalized for the number of estimated parameters. For comparisons of models with different covariates, the more parsimonious model with the smaller BIC is preferred (Raftery 1995). Raftery’s widely cited criteria define “very strong” evidence for a preferred model as BIC difference > 10 (analogous to *p* ≤ 0.01), “strong” evidence = 6–10 (analogous to *p* ≤ 0.05), “positive” evidence = 2–6, and “weak” evidence = 0–2 (Raftery 1995, p. 139).

We conducted sensitivity analysis by comparing HVI predictions of heat deaths with predictions of an alternative indicator of social vulnerability that has been used to identify neighborhoods that are intergenerational “poverty traps.” The index of concentrated disadvantage (Sampson 2009) comprises six neighborhood-level census variables: percentages of individuals who are ethnic minorities, unemployed, or < 18 years of age and percentages of households that are below the poverty line, receiving welfare, or female-headed. HVI and concentrated disadvantage tap a similar underlying construct of neighborhood social disadvantage, but they have only ethnicity and poverty variables in common. The other four variables in the concentrated disadvantage index are not known heat risk factors.

In order to assess whether the 24 July 2000 Landsat scene represented a stable pattern of variability in regional temperatures, we also estimated models with Landsat-based surface temperatures recorded on two alternative dates.

Finally, we used spatial statistics to measure clustering of neighborhoods with similar HVI index scores. We calculated the local indicator of spatial association (LISA; Anselin 1995) to measure spatial autocorrelation of the index. LISA scores for each block group represent the locally disaggregated component of the global Moran’s I (de Smith et al. 2006).

## Results

Results of the principal components analysis are reported in Table 2, which shows the HVI variables loadings on three principal factors: *a*) socioeconomic vulnerability, *b*) elderly/isolation, and *c*) unvegetated area. In contrast with Reid et al. (2009), who identified AC as a separate principal factor for their national HVI, AC/cooler loaded with socioeconomic variables in our analysis. In addition, unvegetated area was an independent factor in Maricopa County, but it was a component of the social/environmental factor in the national HVI. Another difference was that the variable ≥ 65 years of age loaded with diabetes prevalence to form an elderly/diabetes factor in Reid’s national analysis (Reid et al. 2009), whereas in our study, advanced age was a component of the elderly/isolation factor, along with living alone and the interaction between age and living alone. Higher percentages of elderly residents and individuals living alone tended to occur in the same neighborhoods. (We did not include diabetes prevalence in our factor analysis.) Otherwise, except for differences noted above, the Maricopa County HVI factor structure was similar to the factor structure for the national HVI derived by Reid et al. (2009).

**Table 2:** Principal components analysis of heat vulnerability variables in the 2000 U.S. Census Maricopa County block groups (*n* = 2,081).

Variable	Factor loading		
	Factor 1: socioeconomic vulnerability	Factor 2: elderly/isolation	Factor 3: unvegetated area
Ethnic minority	0.91	–0.25	0.04
Latino immigrant	0.90	–0.11	0.06
< Poverty line	0.86	0.04	0.09
No HS diploma	0.92	0.03	0.06
No central AC/cooler	0.79	–0.03	0.09
≥ 65 years of age	–0.19	0.88	–0.04
≥ 65 years of age × living alone	–0.03	0.96	–0.02
Living alone	0.01	0.77	0.01
Unvegetated area (mean)	0.14	0.06	0.91
Unvegetated area (SD)	0.05	–0.10	0.92
HS, high school. Factor extraction was performed using varimax rotation so that the factors are uncorrelated with each other. The numbers in the columns are factor loadings that represent correlations between the variables and factors and also the weights of each variable on the factors.			

**Figure 1:**
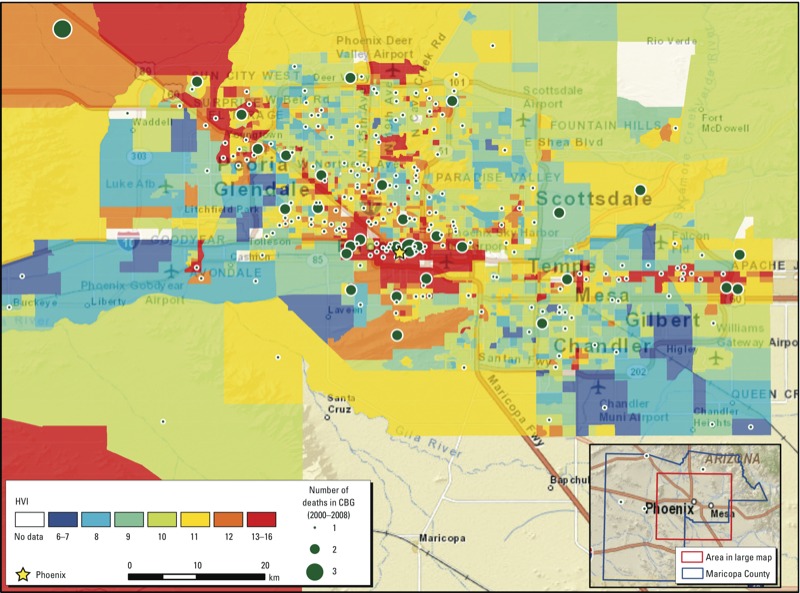
HVI scores (using a method modified from Reid et al. 2009) mapped for 2,081 census block groups (CGBs) in Maricopa County, Arizona. Higher scores represent higher vulnerability. The map inset in the lower right corner indicates the urbanized area of Maricopa County (red box) shown in the larger map. The county, which also contains a much larger area of uninhabited desert and sparse settlement, is outlined in blue. The urbanized area covers all the cities and all but one of the major towns in the county. Residences of only four people who died from heat exposure were located outside the urbanized area (green circles in inset).

The map of metropolitan Phoenix (Figure 1) illustrates HVI integer scores for block groups from lowest to highest vulnerability. Neighborhoods in the inner cores of the two largest cities (Phoenix and Mesa) and along a corridor in the northwestern suburbs (Glendale to Sun City) had the highest scores. Lowest scores were in urban fringe neighborhoods to the east and west of major municipal centers. The distribution of heat deaths (Figure 1) shows that residential neighborhoods of decedents who died from heat exposure were located throughout the metropolitan area, but block groups with two or three deaths were more common in higher vulnerability areas.

Results are reported in Table 3 for four binary logistic regression models predicting the odds of heat exposure deaths for neighborhood residents. The predictors were HVI integer score of block group (model 1), three normalized factor scores (model 2), surface temperature mean and SD (model 3), and two factors plus surface temperature (model 4). *p*-Values were < 0.001 for population size in each model (not shown). Hosmer-Lemeshow (Hosmer et al.1991) chi-square statistics showed that goodness-of-fit for each model was adequate (*p*-values ≥ 0.46).

**Table 3:** ORs (95% CIs) from binary logistic regression models of at least one heat-associated death in the 2000 U.S. Census Maricopa County block group of residence, 2000–2008^*a*^ (*n* = 2,081).

Variable	Model 1: HVI integer scores	Model 2: HVI factor scores 1–3	Model 3: land surface temperature on 24 July 2000	Model 4: HVI factor scores 1 and 2 and land surface temperature
HVI (integer scores)	1.34** (1.23, 1.45)
Socioeconomic vulnerability (HVI factor 1)		1.50** (1.33, 1.70)		1.34** (1.17, 1.53)
Elderly/isolation (HVI factor 2)		1.38** (1.22, 1.56)		1.39** (1.23, 1.56)
Unvegetated area (HVI factor 3)		1.19* (1.02, 1.39)
Land surface temperature (mean)			1.32** (1.20, 1.45)	1.23** (1.11, 1.36)
Land surface temperature (SD)			1.16* (1.01, 1.34)	1.09 (0.94, 1.27)
–2 log L	1406.64	1385.18	1412.40	1372.73
Hosmer-Lemeshow p-value	0.46	0.99	0.74	0.58
BICb	1429.56	1423.38	1442.96	1418.57
Abbreviations: CI, confidence interval; OR, odds ratio. Dependent variable: at least one decedent who died from heat exposure lived in the census block group (1 = yes; 0 = no). aIntercept and population size of census block groups in each model; p < 0.001 (not shown). bBIC = -2logL+Np*Ln(n) where Np = number of parameters and n = 2,081. *p ≤ 0.05. **p ≤ 0.001.				

In models 1–3, each social and biophysical neighborhood indicator was associated with the odds of one or more deaths from heat exposure among census block residents (*p*-values < 0.05) (Table 3). In model 3, a 1^o^C increase in mean surface temperature was associated with a 32% increase in the odds of a death from heat exposure, after adjusting for surface temperature SD and population size.

Using the BIC and Raftery’s (1995) statistical criteria for goodness-of-fit model comparison, there was positive evidence that model 2 (HVI factor scores) is preferred over model 1 (HVI additive index score). There was very strong evidence that model 2 is preferred over model 3 (only surface temperature) and positive evidence that model 4 (socioeconomic vulnerability and elderly/isolation factors and surface temperature) is preferred over model 2.

The other four HVI models we tested used each factor score and the percent no AC/cooler variable one at a time (results not shown). Using Raftery’s (1995) criteria for comparing BICs, there was strong to very strong support for preferring models 1, 2, and 4 over models with only single factors or no AC/cooler; strong to very strong support for preferring model 3 over models with only elderly/isolation, unvegetated area, or no AC/cooler; and positive support for preferring a model with the socioeconomic vulnerability factor over model 3.

The model with the index of concentrated disadvantage had a poor goodness-of-fit statistic (Hosmer-Lemshow *p*-value = 0.098, results not shown). We substituted regional surface temperatures from Landsat scenes on 2 other days into models 3 and 4 [see Supplemental Material, Table S1 (http://dx.doi.org/10.1289/ehp.1104625)]. Estimates based on regional temperatures on 6 June 2000 were nearly identical to those for 24 July 2000, but estimates based on data for 25 October 1990 were attenuated, which is consistent with smaller differences in vegetation between neighborhoods due to lower heat stress in autumn compared with the summer months. (Almost no heat exposure deaths occur in October.)

**Figure 2:**
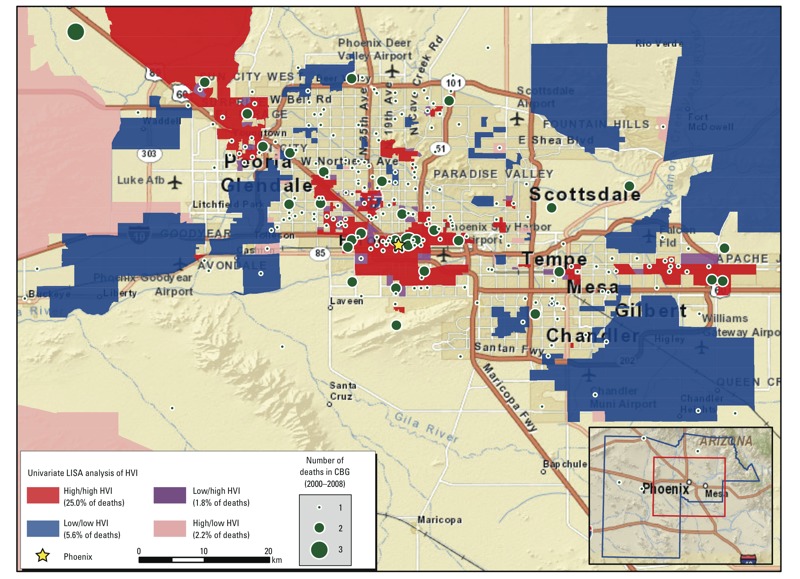
Univariate analysis of the LISA-identified clusters of census block groups (CBGs) in Maricopa County, Arizona, with similar or dissimilar HVI scores (*p*-value ≤ 0.05). High/high areas in the map are clusters of neighboring CBGs with uniformly high vulnerability scores; low/low areas are clusters with low vulnerability scores; low/high areas represent a CBG with a low vulnerability score neighbored by high vulnerability CBGs; high/low areas represent a CBG with a high vulnerability score neighbored by low vulnerability CBGs. Entries in the legend (next to the colored boxes) also show the percentages of 2000–2008 heat-related decedents who were residents in each type of cluster.

We observed a moderate degree of overall spatial clustering of vulnerability scores among neighborhoods (Global Moran’s I for HVI = 0.37; *p* = 0.05). LISA analysis (Figure 2), identified neighborhood clusters with similar or dissimilar scores (*p* ≤ 0.05). High/high (red) clusters in Figure 2 represent neighborhoods with extremely high vulnerability scores next to others with extremely high scores. One-quarter of heat decedents lived in high/high clusters. Only 9.6% of heat decedents lived in clusters with low/low vulnerability scores (blue) or dissimilar scores (e.g., purple areas, indicating low vulnerability neighborhoods next to high). Sixty percent of the 50 heat deaths among homeless people occurred in high/high vulnerability clusters [see Supplemental Material, Figure S1 (http://dx.doi.org/10.1289/ehp.1104625)].

## Discussion

We evaluated socio-environmental influences on heat-associated deaths in this study. Mapping and statistical analyses were used to identify “hot spots” of vulnerable places where socioeconomic disadvantages, unvegetated landscapes, and high temperatures were collocated with residences of heat decedents. Our analysis extended methods used in a small number of previous studies that identified neighborhoods at risk for heat-related deaths.

The factor structure of a heat vulnerability index derived for Maricopa County was generally consistent with an index developed for the nation by Reid et al. (2009). Two exceptions were the configurations of factor loadings for AC and vegetation variables in our principal components analysis that may be particular to desert cities. Only 13% of single-family homes in the Phoenix area lacked central AC or an evaporative cooler, which is much lower than a cross-section of 14 other U.S. cities (Janssen et al. 2002). The desert southwest has an exceptionally hot climate and only the poorest households in Phoenix go without AC or a cooler. In our study, AC had a strong negative correlation with neighborhood poverty, low educational attainment, and proportions of ethnic minorities and Latino immigrants (Table 1). All these variables loaded on the same factor, suggesting that AC in single-family homes is related to socioeconomic vulnerability to heat in Maricopa County (Table 2).

Unvegetated area was a separate factor in our analysis and had a weak but significant positive association with the odds of at least one heat death in a census block. These findings indicate that vegetation may be a protective factor against human heat stress in all neighborhoods in desert cities. Our study confirms the importance of understanding regional variations in heat vulnerability indicators.

The preferred model for predicting residents’ odds of dying from heat exposure in Maricopa County used socioeconomic vulnerability and elderly/isolation factors and mean surface temperature adjusted for population size and surface temperature SD in residential neighborhoods. The HVI model was preferred over a model using an index of neighborhood concentrated disadvantage, which included two social variables that are specifically related to heat risk and four variables that are not. Measurement errors in AC, land cover, and temperature may have attenuated the relationships between some vulnerability indicators and heat deaths. Given strong interest in adapting local built environments to climate change, it is important to continue developing more refined measures of environmental variables.

We used decedents’ residential addresses as the indicator of vulnerable places because *a*) they represent the social and ecological contexts of many daily activity spaces, *b*) they are consistent with other studies of neighborhood effects on social and health outcomes, and *c*) the place of injury address on death certificates is not consistently coded for heat-related deaths. Mapping heat deaths onto the HVI derived for our study population showed that one-quarter of decedents lived in a relatively small number of highly vulnerable neighborhood clusters that were mainly in central city areas (red areas in Figure 2). Living conditions could have contributed to these deaths but people might have died in a different place, such as at work or other activity sites. More research is needed on the circumstances of heat-related deaths.

We can say, however, that a majority of heat deaths among the homeless were reported in high vulnerability areas. Homeless people live in areas near downtown Phoenix and along industrial and transportation corridors that extend east and northwest from the central city where shelters, services, and hospitals are located (Sanchez 2011).

Heat mortality research in urban neighborhoods should be replicated in other areas with different urban forms and climates. More research is also needed to understand processes underlying the spatial distribution of heat-vulnerable places and their relationships to deaths, especially deaths from other causes that may be related to hot weather, such as cardiovascular disease (McMichael et al. 2006). We did not include a neighborhood indicator of general population health or specific disease incidence in our analysis but if such an indicator were available, it might be an important vulnerability marker. In addition, the deaths we analyzed were identified by the county’s heat death surveillance system, which could introduce unknown sources of variation into how causes of deaths were identified. Weather and other circumstances beyond the scope of this study could affect the temporal and spatial distributions of deaths over 9 years.

This type of research provides a foundation of knowledge for local interventions. We compared several ways that models could be constructed using census data and remotely sensed data available for the United States. Surface temperature may be a reasonable substitute for indices that rely on statistical computations with many variables if the intended purpose of the model is to prevent heat-related deaths. Although model 4, which included several population characteristics and temperature, was preferred for predicting the odds of heat deaths, model 3, which only included temperature variables, also had an adequate goodness-of-fit statistic. Temperature difference is a simple way to communicate relative risks to residents of vulnerable neighborhoods. Satellite data relevant to neighborhood-scale investigations has excellent spatial coverage, but it is collected discontinuously. Augmentation with a network of weather stations to measure air temperatures could also be useful.

There are many ways in which health interventions could be targeted to heat-vulnerable neighborhoods: extreme weather warning systems, preventive actions for the homeless, improved emergency response, or heat island mitigation. Although AC has been widely associated with lower morbidity and mortality rates from heat-related illnesses, it is not a panacea for adapting to rising urban temperatures and heat waves. Vulnerable people and places do not have equal access to AC in Maricopa County, in other cities (Janssen et al. 2002; O’Neill et al. 2005), or in developing countries with climates hotter than Phoenix (Sivak 2009). Looking toward the mid-to-late 21st century, the southwestern United States is expected to experience continuing drought that may dramatically reduce the amount of water available to irrigate urban vegetation for cooling outdoor temperatures and to produce energy for powering AC (Gober 2010). Over 90% of Arizona’s energy is supplied by coal, natural gas, and nuclear power plants that use large amounts of water in the production and delivery of electricity (Pasqualetti and Kelley 2007). The complete array of responses needed to reduce heat risks for people in vulnerable places is complex and public health officials should cooperate with other stakeholders to coordinate a broad range of policies.

## Conclusions

We estimated the neighborhood effects of population characteristics and features of the built and natural environments on deaths due to heat exposure in Maricopa County, Arizona (2000–2008). Spatial patterns showed substantial variability between neighborhoods in vulnerability to heat, odds of residents dying from heat exposure, and locations of vulnerable neighborhood clusters. Many inner-city neighborhoods had higher vulnerability scores and more deaths, whereas higher neighborhood income and education, younger white populations, greener landscapes, AC, and cooler microclimates in suburban neighborhoods were associated with reduced heat vulnerability and fewer deaths. Heat deaths of homeless persons were reported primarily in the inner city. Many decedents, however, lived in neighborhoods with lower vulnerability scores and, therefore, place-based indicators of vulnerability are complements and not substitutes for person-level risk variables. Surface temperature might be used as a single indicator in Maricopa County to identify the most heat-vulnerable neighborhoods. However, more attention to the socioecological complexities of climate mitigation and adaptation is a high public health priority. There are major local challenges ahead in preventing heat-related deaths under global regimes of climate change and urbanization.

## Supplemental Material

(610 KB) PDF
