# Supplementary material for: Neighborhood Effects on Heat Deaths: Social and Environmental Predictors of Vulnerability in Maricopa County, Arizona
Source: Environ Health Perspect. 2012 Nov 16;121(2):197–204. doi: 10.1289/ehp.1104625 (PMC3569676; doi:10.1289/ehp.1104625)
Supplement: (610 KB) PDF [file ehp.1104625.s001.pdf]

Supplemental Material

Title: Neighborhood Effects on Heat Deaths: Social and Environmental Predictors of Vulnerability in Maricopa County, Arizona

Authors: Sharon L. Harlan, Juan H. Declet-Barreto, William L. Stefanov, Diana B. Petitti

Page 2: Table S1

Page 3, 4: Figure S1

Table S1. Binary logistic regression models using land surface temperature on two dates to predict odds of 2000-2008 heat-associated deaths in the 2000 U.S. Census Maricopa County block group of residence [a, b] (n=2,081)

Date	Land Surface Temperature 06/06/2000				Land Surface Temperature 10/25/1990			
	Model 3 Land Surface Temperature		Model 4 HVI Factor Scores 1 and 2 and Land Surface Temperature		Model 3 Land Surface Temperature		Model 4 HVI Factor Scores 1 and 2 and Land Surface Temperature	
Variables in Models	Odds ratio	CI[c]	Odds ratio	CI	Odds ratio	CI	Odds ratio	CI
Socioeconomic Vulnerability (HVI Factor 1)			1.32***	(1.15,1.51)			1.49***	(1.32,1.70)
Elderly/Isolation (HVI Factor 2)			1.40***	(1.24,1.59)			1.41***	(1.25,1.60)
Land Surface Temperature (mean)	1.30***	(1.19,1.42)	1.22***	(1.11,1.35)	1.04	(0.96,1.13)	1.09	(0.99,1.19)
Land Surface Temperature (SD)	1.11	(0.98,1.26)	1.05	(0.92,-1.20)	0.88	(0.73,1.06)	0.95	(0.78,1.14)
-2 log L	1411.37		1371.42		1446.55		1386.54	
Hosmer-Lemeshow p-value	0.66		0.31		0.58		0.78	
BIC[d]	1441.93		1417.26		1477.12		1432.39	

a. Dependent variable: at least one decedent who died from heat exposure lived in the census block group (1=yes; 0=no).
b. Intercept and population size of census block groups in each model; p<0.001 (not shown).
c. CI: confidence interval.
d. BIC = -2logL+Np*Ln(n) where Np=number of parameters.

* p<=0.05; ** p<=0.01; *** p<=0.001

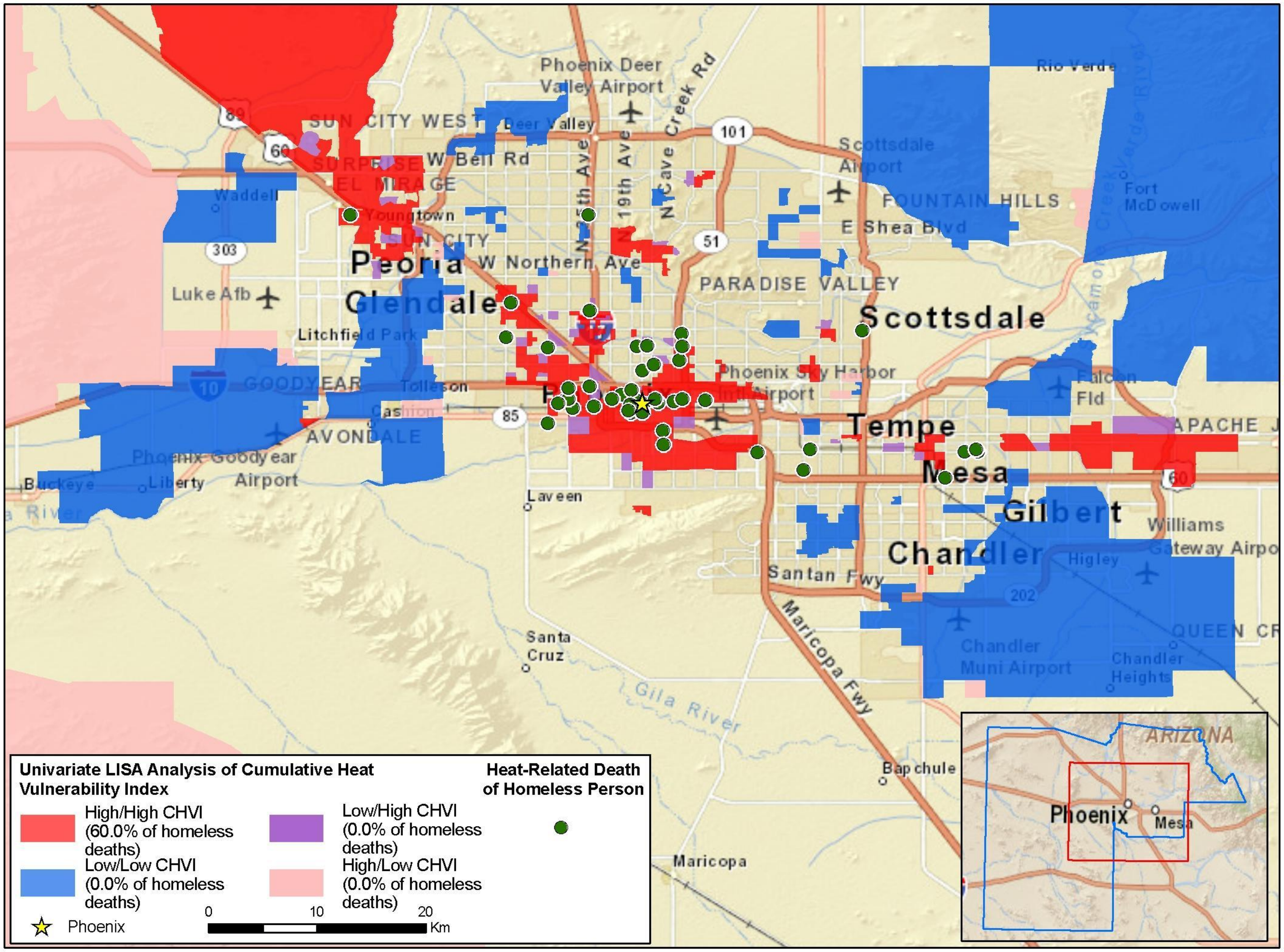

Figure S1. Heat-associated deaths of homeless persons in Maricopa County, AZ (2000-2008). Univariate analysis of the Local Indicator of Spatial Association (LISA) shows clusters of census block groups in Maricopa County, AZ with similar or dissimilar Heat Vulnerability Index scores

Figure S1 (continued)
(p-value<=.05). High/high (red) areas in the map are clusters of neighboring census block groups with uniformly high vulnerability scores; low/low (blue) areas are clusters with low vulnerability scores; low/high (purple) areas represent a census block group with a low vulnerability score neighbored by high vulnerability block groups; high/low (pink) areas represent a census block group with a high vulnerability score neighbored by low vulnerability block groups. Circles on the map indicate locations of heat-associated deaths among homeless persons (2000-2008) in block groups where death was reported (place of injury field on the death certificate). The definition of homeless person is a resident of Maricopa County with unknown residential street address on the death certificate. Sixty percent of the 50 heat deaths among homeless persons were reported in high/high vulnerability clusters.
